# Method of Shrinking Human Heads by Baqueta Indians

**Published:** 1917-07

**Authors:** Horacio Giraloto

**Affiliations:** Palmeiro, Brazil


### Method of Shrinking Human Heads by Baqueta Indians.

REV. HORACIO GIRALOTO, Palmeiro, Brazil.

Translation by Major W. W. Quinton, M. D.

During a voyage on the River Maranon I had occasion to converse with a person who had been present at the operation by means of which the Baqueta Indians reduce the size of the heads they cut from their prisoners of war, drying them out instantly and rendering them incorruptible, (i. e. not subject to decay).

This operation generally is intrusted to the women and is as follows:

On a well-cleaned floor they commence to construct a small oven of clay in which can be contained comfortably the head they wish to prepare.

To construct this oven they make in the first place a framework with the twigs of trees and over this place a covering of clay in such a manner that the branches are covered, leaving only a small opening at the bottom and six holes in the vault of the oven.

On the outside and all around the oven they light six fires, over each one of which they place a jar of water. This jar is sealed hermetically except that from the lid of each one of these jars issues a tube of clay that rises to the holes in the lid and penetrates the oven, conducting the steam from the jar to the oven.

When everything is ready and the fire is lighted, they place in the oven the head that they wish to prepare and when the water is boiling and the steam commences to pass in the tubes they shut up with clay the opening of the oven.

The fire is fed for 8 hours more or less and the Indians renew the water in the jars which has been lost by evaporation.

After 8 hours they open the oven, extract the head which they find much reduced in size and place it to dry in the sun and the operation is finished.

According to repeated assurances made to me they practice no manipulation nor pressure over the head nor remove any of the brain or the skull.

The prolonged action of the steam seems to be the only agent in this curious operation.

